# Prognostic value of tumour volume based on [^18^F]PSMA-1007 PET/CT in prostate cancer

**DOI:** 10.1186/s41824-026-00292-w

**Published:** 2026-03-13

**Authors:** Jacob Ingvar, Johan Wittgren, Vigdis Sverrisdottir, Elin Trägårdh, Anders Bjartell

**Affiliations:** 1 Department of Urology, Skåne University Hospital, Jan Waldenströmsgata 5, Malmö, 205 02 Sweden; 2 Department of Translational Medicine, Lund University, Malmö, Sweden; 3 Clinical Physiology and Nuclear Medicine, Skåne University Hospital, Malmö, Sweden; 4 Wallenberg Centre for Molecular Medicine, Lund University, Lund, Sweden

**Keywords:** PET/CT, Prostate cancer, PROMISE, TLV, Overall survival

## Abstract

**Background:**

Prostate-specific membrane antigen positron emission tomography/computed tomography (PSMA PET/CT) enables sensitive detection and staging of prostate cancer, yet its relationship with long-term survival remains unclear. This study aimed to assess whether total lesion volume (TLV) measured on [^18^F]PSMA-1007 PET/CT predicts overall survival (OS) in prostate cancer patients.

**Methods:**

A cohort of 282 patients scanned between 2019 and 2021 was retrospectively studied, including both patients undergoing primary staging and imaging for biochemical recurrence (BCR). Tumour lesions in the prostate/prostate bed, lymph nodes and bone were manually segmented and TLV was calculated for each compartment and as total of all lesions. Based on TLV, patients were categorized into three groups for each compartment: no detectable tumour, TLV ≤ median, and TLV > median. Bone analyses were dichotomised into any vs. no detectable tumour. The association with OS was assessed using age-adjusted Cox proportional-hazards models and Kaplan–Meier analysis, separately for the primary staging subgroup and the BCR subgroup.

**Results:**

Over a mean follow-up of almost 5 years, 49 patients (17%) died. In the primary staging subgroup (*n* = 171), detectable bone metastases on PSMA PET-CT predicted poorer survival compared to no detectable bone metastasis (HR 2.39, *p* = 0.031). Among patients with BCR (*n* = 111), total TLV above the median and a prostate TLV above the median both predicted an almost 5 times increased risk of death.

**Conclusion:**

Total lesion volume on [^18^F]PSMA-1007 PET/CT was associated with OS, with different prognostic patterns in primary staging and BCR. The results support future incorporation of volumetric PSMA PET metrics into personalized staging and therapeutic decision-making.

**Supplementary Information:**

The online version contains supplementary material available at 10.1186/s41824-026-00292-w.

## Introduction

Prostate-specific membrane antigen positron emission tomography/computed tomography (PSMA PET/CT) was introduced over a decade ago and has since gained widespread recognition as a highly accurate imaging modality for the detection and staging of prostate cancer. Compared to conventional imaging techniques such as CT and bone scintigraphy (BS), PSMA PET/CT has consistently demonstrated superior sensitivity and specificity in identifying prostate cancer lesions (Hofman et al. 2020; Mazzone et al. 2025). This imaging modality has proven beneficial across the entire disease spectrum—from initial staging to biochemical recurrence (BCR) and metastatic disease (Fendler et al. 2019). In addition, stratification into low or high volume of disease based on conventional imaging (CI) has been shown to be strongly associated with outcome in prostate cancer patients (Francini et al. 2018; Shiota et al. 2021).

Although PSMA PET/CT is superior in sensitivity for detecting metastases, the relationship between PSMA PET/CT findings and long-term survival outcomes has not been thoroughly explored or integrated into clinical decision-making.

According to the EAU guidelines (Herrmann et al. 2025), PSMA PET/CT is currently recommended for use in cases of BCR. While the guidelines state that PSMA PET/CT provides more accurate staging of high-risk prostate cancer compared to CI it has not yet been shown to more accurately predict survival.

Some studies have shown that PSMA PET/CT–derived total tumour volume is a prognosticator of overall survival (OS) in patients with end-stage disease (Emmett et al. 2025) while other studies have demonstrated conflicting results (Telli et al. 2025).

A recently published study from Emmett et al. (Emmett et al. 2025) on patients treated with the addition of [^177^ Lu]Lu-PSMA-617 to enzalutamide as first-line treatment for high-risk prostate cancer showed that baseline total lesion volume in PSMA-PET/CT is prognostic for OS and predictive for a beneficial effect on OS but PSMA mean standardized uptake value (SUVmean) was not prognostic for PSA progression-free survival or OS when [^1 7 7^ Lu]Lu-PSMA-617 was administered with enzalutamide.

Newly published recommendations from the Standardised PSMA PET/CT Analysis and Reporting Consensus (SPARC) highlight that PSMA volume, along with SUVmean and maximum SUV (SUVmax), can be measured reliably, with PSMA volume recognized as a quantifiable prognostic biomarker across different stages of prostate cancer (Herrmann et al. 2025).

In order to bring uniformity and clarity to the interpretation of PSMA PET/CT scans, the Prostate Cancer Molecular Imaging Standardized Evaluation (PROMISE) framework was introduced in 2018 (Eiber et al. 2018) and later updated in 2023 (Seifert et al. 2023). PROMISE provides a structured system for categorizing PSMA PET/CT findings into clinically relevant stages and includes parameters such as SUVmean. While PROMISE has been instrumental in standardizing scan interpretation and disease characterization, it does not currently incorporate prognostic metrics related to OS.

A recent retrospective, multicentre study by Karpinski et al. in 2024 aimed to address this gap by evaluating the prognostic utility of PSMA PET/CT (Karpinski et al. 2024). The study analyzed data from 2414 prostate cancer patients who underwent PSMA PET/CT at various stages of their disease. Using the PROMISE framework for scan interpretation, the results demonstrated that tumour volume was predictive of OS across both early and late stages of disease.

The aim of this study was to assess if tumour burden, quantified as total lesion volume (TLV) on [^18^F]PSMA-1007 PET/CT is associated with OS in patient with prostate cancer. To reflect distinct clinical scenarios, we analyzed two separate patient populations: men undergoing primary staging and men with biochemical recurrence after prior treatment with curative intent. For both cohorts, TLV was assessed in four anatomical compartments: prostate/prostate bed, lymph node metastases, bone metastases, and total TLV. This was done to determine their individual and combined prognostic value.

## Material & methods

### Patient cohort

We retrospectively evaluated a consecutive series of patients who underwent [^18^F]PSMA-1007 PET/CT examination for either primary staging of unfavorable intermediate risk and high-risk disease, or BCR of prostate cancer, from September 2019 to May 2021 at Skåne University Hospital in Southern Sweden. Data was extracted from medical records included age, date of diagnosis, treatment modality, PSA, Gleason score, results of PSMA PET/CT and details from pathology reports.

The study protocol was approved by the Regional Ethics Committee in Lund (#2016/417, #2018/117, #2018/753, #2020–02524), in adherence to ethical standards in accordance with the Declaration of Helsinki. Informed consent was obtained from all participants prior to inclusion in the study.

### PET/CT imaging and tumour segmentation

Patients were injected with 4 MBq/kg [^18^F]PSMA-1007 and underwent a PET/CT scan from the base of the skull to the mid-thighs on a GE Discovery MI PET-CT system (Discovery MI; GE Healthcare, Milwaukee, WI, USA) 120 minutes post-injection. Data were reconstructed using the Q.Clear (GE Healthcare, Milwaukee, WI, USA) algorithm with a β-value of 800, including time-of-flight, point spread function and CT-based attenuation correction with a 256 × 256 matrix (pixel size 2.7 × 2.7 mm, slice thickness 2.8 mm).

Tumour segmentation was performed using the cloud-based RECOMIA platform (http://www.recomia.org), as previously described (Tragardh et al. 2020). All lesions with visually suspicious uptake were first manually annotated by an experienced nuclear medicine physician, independent of SUV intensity. After manual segmentation, thresholding was applied according to the method described by Gafita et al. (Gafita et al. 2022). These thresholds were used to calculate TLV within each annotated region. As a consequence, some manually segmented lesions with SUV below the threshold were automatically excluded from volume calculation, whereas other lesions changed in measured volume when the threshold was applied.

### Statistical analysis

Analyses were performed separately for patients assessed for primary staging and BCR as well as combined. The analysis was further divided into specific categories: TLV of bone metastasis, TLV of lymph node metastasis, and TLV of prostate lesion/local recurrence, as well as total TLV. Furthermore, the TLV was categorized into three groups: no detectable tumour, a TLV smaller than the median, and a TLV greater than the median. Given the low sample sizes in the bone metastasis categories, all patients with detectable bone metastasis were collapsed into one group. The median, mean, interquartile range (IQR), and total range for TLV were calculated for total TLV, bone TLV, lymph node TLV and prostate TLV.

OS was the primary outcome of interest and defined as time from PSMA-PET/CT to death from any cause. We extracted information from the National Cause of Death Registry regarding incident deaths. Censoring events were either occurrence of death or the end of the study by January 3, 2025.

Survival-analysis were estimated using Cox proportional-hazards models and Kaplan–Meier analyses. The models were adjusted for age. Multivariable adjustment beyond age was not performed due to the limited number of deaths within each subgroup. Patients with no uptake on the PSMA-PET/CT were used as reference group. Hazard ratios (HR) and 95% confidence intervals (CI) are presented. The significance level was set to 0.05. Analyses were performed using R version 4.3.3.

## Results

### Patient demographics and follow-up

In total, 282 patients were included in the analysis of OS, with 171 in the staging group and 111 in the BCR group. At the end of follow-up, 49 patients (17%) had died. The average follow-up period was 5.0 years. Patient characteristics are summarized in Table 1. The distribution of patients of the different TLV-categories is shown in Table 2. Results from the combined staging and BCR cohorts are presented in supplementary Table 1 and supplementary Figure 1. Combined analyses are provided as supplementary material for completeness but were not used for inference due to cohort heterogeneity.

**Table 1:** Patient characteristics

Patient- and tumour characteristics	All analysis	Staging subgroup	BCR subgroup
Total number of patients, n (%)	282 (100)	171 (61)	111 (39)
Age (mean, range)	71 (42–87)	70 (42–87)	71 (47–83)
Number of deaths, n (%)	49 (17)	30 (18)	19 (17)
Follow-up time (mean years, range)	5.0 (0.1–5.3)	4.9 (0.1–5.3)	5.0 (0.4–5.3)
D’Amico risk group, n (%)			
Low	5 (2)	0 (0)	5 (5)
Intermediate	93 (35)	39 (23)	54 (57)
High	164 (63)	128 (77)	36 (38)
Missing	20	4	16

**Table 2:** Distribution of patients in the analyses for primary staging and biochemical recurrence (BCR)

	All patients	Patients in primary staging analyses	Patients in BCR analyses
Total TLV, n (%)			
0	55 (20)	9 (5)	46 (41)
≤ median	114 (40)	73 (43)	41(37)
> median	113 (40)	89 (52)	24 (22)
Bone TLV, n (%)			
0	228 (81)	137 (80)	91 (82)
≤ median	27 (10)	17 (10)	10 (9)
> median	27 (10)	17 (10)	10 (9)
Lymph node TLV n (%)			
0	196 (70)	129 (75)	67 (60)
≤ median	43 [15]	24 (14)	19 (17)
> median	43 [15]	18 (11)	25 (23)
Prostate TLV, n (%)			
0	95 (34)	13 (7)	82 (74)
≤ median	94 (33)	71 (42)	23 (21)
> median	93 (33)	87 (51)	6 (5)

The calculated total TLV in the primary staging cohort had a mean of 24.9 ml and a median of 5.1 ml. In the BCR cohort, the mean and median TLV were 22.8 ml and 2.0 ml, respectively for total TLV. For the different locations the calculated TLV including median, mean, interquartile range (IQR), and total range are presented in Tables 3 and 4 for primary staging and BCR separately.

**Table 3:** TLV (ml) in the primary staging group

	Mean	Median	Min	Max	Q1	Q3
Total TLV	24.9	5.1	0.1	1307.1	2.5	11.2
Bone TLV	30.9	2.6	0.3	541.5	1.1	12.3
Lymph node TLV	10.9	0.8	0.0	161.4	0.2	3.8
Prostate TLV	16.0	4.1	0.1	616.9	2.2	10.4

Q1 – 25^th^ percentile; Q3 – 75^th^ percentile

**Table 4:** TLV (ml) in the BCR group

	Mean	Median	Min	Max	Q1	Q3
Total TLV	22.8	2.0	0.0	422.6	0.6	10.6
Bone TLV	32.7	2.7	0.3	421.0	0.6	14.0
Lymph node TLV	14.7	1.2	0.0	173.0	0.5	11.6
Prostate TLV	6.3	1.0	0.0	54.0	0.3	3.2

Q1 – 25^th^ percentile; Q3 – 75^th^ percentile

### Primary staging cohort

**Fig. 1:**
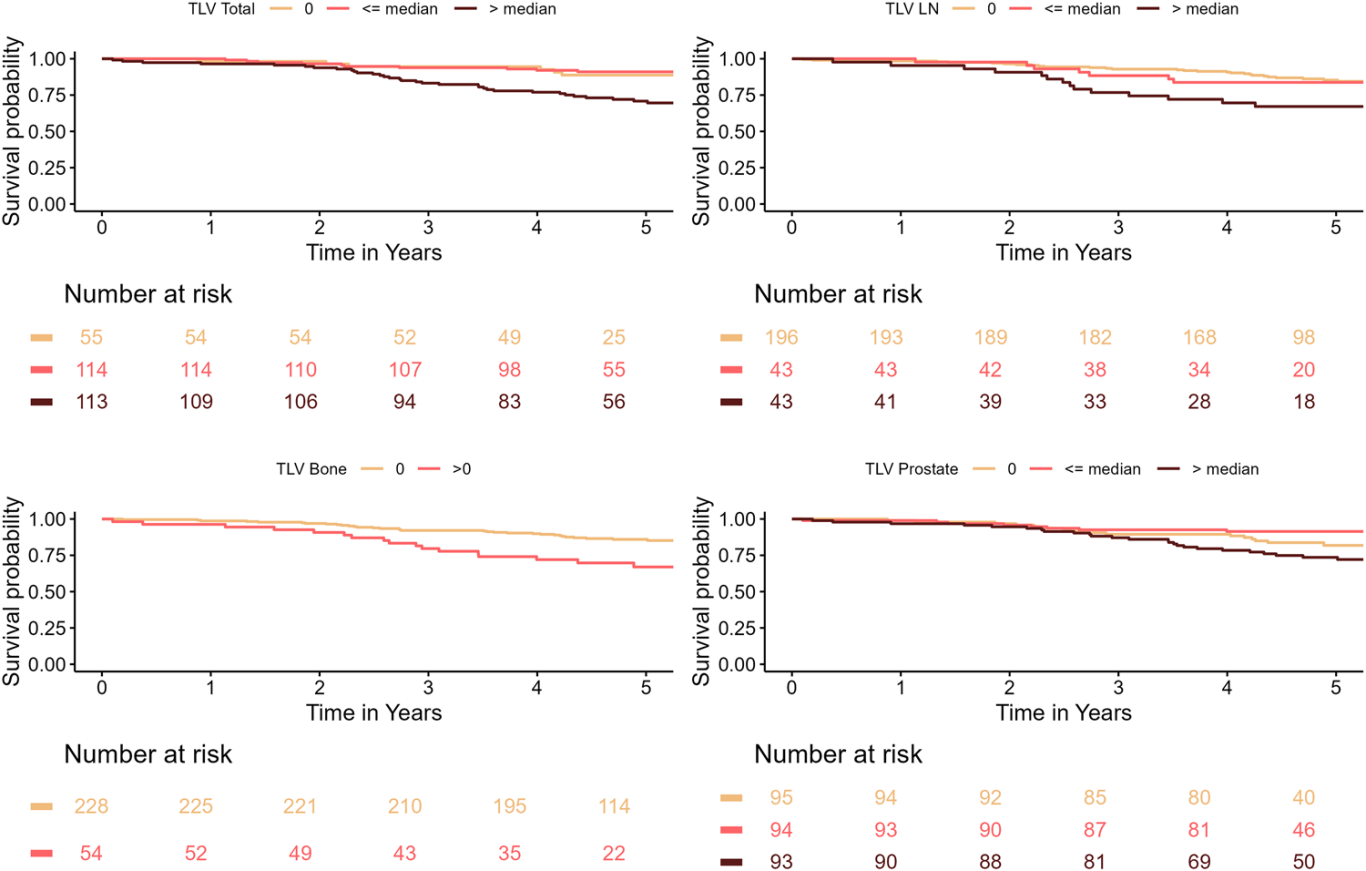
Survival curves for the primary staging cohort by total, lymph node, bone and prostate tumour volume. Tumour volume divided in none, below median, and above median for total TLV, lymph node TLV and prostate TLV. Bone TLV presented as not detectable or detectable

In the adjusted analysis of the primary staging cohort (Table 5), the total TLV was not associated to OS. However, detectable bone metastases on PSMA PET-CT were significantly associated with a worse outcome compared with no detectable bone metastases, with an HR of 2.39 (95% CI: 1.13–5.07) adjusted for age and HR of 2.74 (95% CI: 1.30–5.77) for unadjusted estimates. In the unadjusted analyses for lymph node TLV, a TLV > median was significantly associated with OS (HR 2.99; 95% CI 1.26–7.08), but this association was slightly reduced in the adjusted analyses and became non-significant. A prostate TLV below the median was negatively associated to OS, with an adjusted HR of 0.12 (95% CI: 0.03–0.50), indicating an improved survival for these patients vs. patients with a prostate TLV of 0. For values above the median, no significant association to OS could be detected. Figure 1 shows Kaplan Meier curves for the primary staging cohort.

**Table 5:** Hazard ratios and 95% confidence intervals for overall survival including primary staging patients

Variable	Unadjusted		Adjusted^*^	
	HR (95% CI)	p-value	HR (95% CI)	p-value
Total TLV		0.001		0.002
0	1 (Ref.)		1 (Ref.)	
≤ median	0.23 (0.04; 1.26)		0.19 (0.03; 1.02)	
> median	1.22 (0.29; 5.19)		0.89 (0.20; 3.84)	
Bone TLV		0.012		0.031
0	1 (Ref.)		1 (Ref.)	
>0	**2.74 (1.30; 5.77)**		**2.39 (1.13; 5.07)**	
Lymph node TLV		0.061		0.127
0	1 (Ref.)		1 (Ref.)	
≤ median	0.81 (0.24; 2.71)		0.67 (0.20; 2.28)	
> median	**2.99 (1.26; 7.08)**		2.33 (0.97; 5.59)	
Prostate TLV		0.002		0.002
0	1 (Ref.)		1 (Ref.)	
≤ median	**0.17 (0.04; 0.69)**		**0.12 (0.03; 0.50)**	
> median	0.81 (0.28; 2.37)		0.52 (0.17; 1.57)	

* Adjusted for age. Hazard ratios (HR) and 95% confidence intervals (95% CI) derived from Cox regression analyses. Analyses include patients from the primary staging cohort

### Biochemical recurrence cohort

**Fig. 2:**
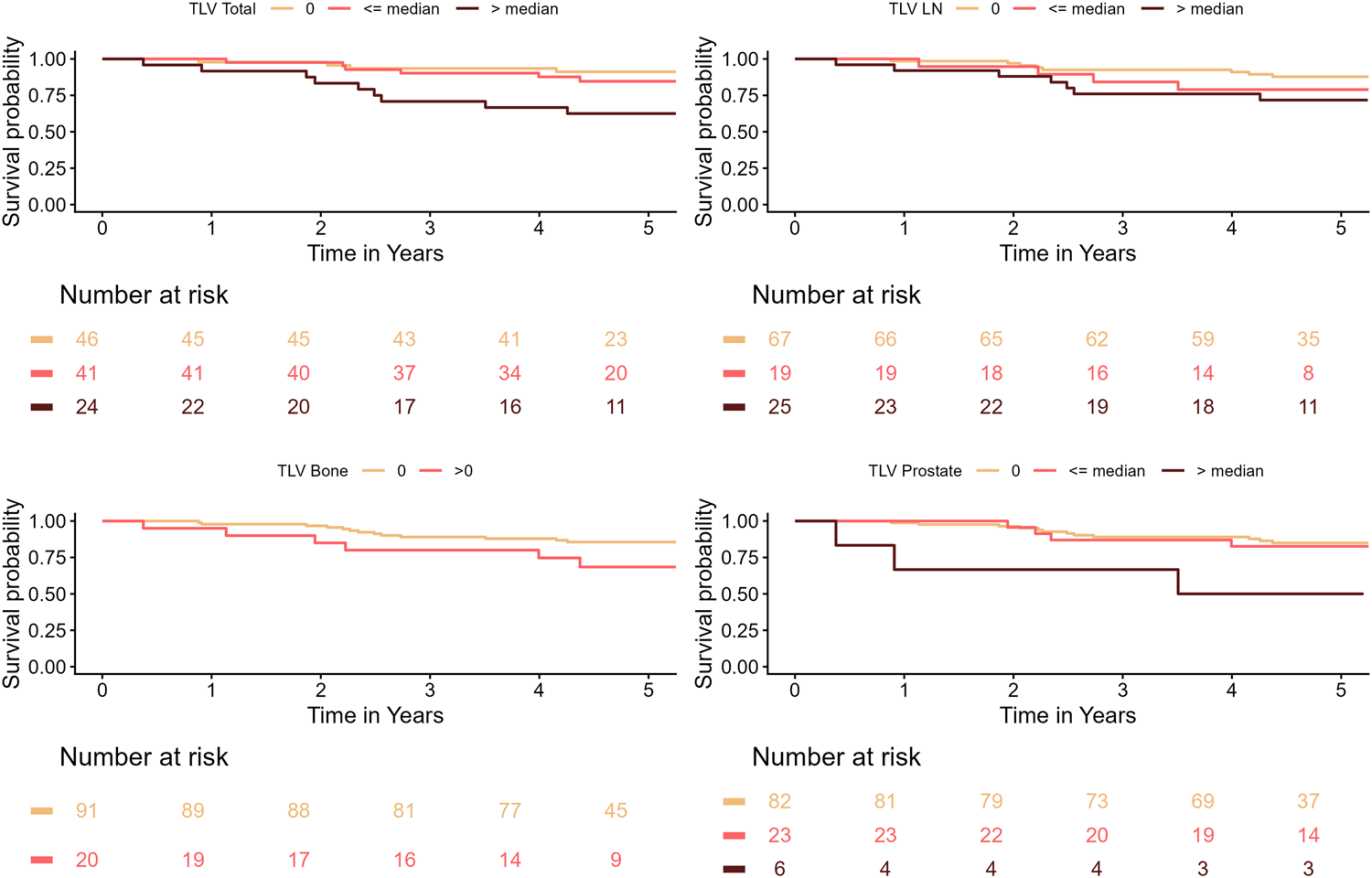
Survival curves for the BCR cohort by total, lymph node, bone and prostate tumour volume. Tumour volume divided in none, below median, and above median for total TLV, lymph node TLV and prostate TLV. Bone TLV presented as not detectable or detectable

In the BCR group (Table 6), total TLV ≤ median was not significantly associated with OS. However, total TLV > median was associated with a 5-times increased risk of death in the unadjusted and age-adjusted analyses (*p* = 0.015 and 0.019). On the contrary, bone TLV > 0 and lymph nodes were not a significant predictor of death. With regards to prostate TLV, a TLV above the median was significantly associated with OS, (HR_adj_ 4.79 (95% CI: 1.35–17.00). Kaplan Meier curves for the BCR cohort are presented in Fig. 2.

**Table 6:** Hazard ratios and 95% confidence intervals for overall survival including biochemical recurrence patients

Variable	Unadjusted		Adjusted^*^	
	HR (95% CI)	p-value	HR (95% CI)	p-value
Total TLV		0.015		0.019
0	1 (Ref.)		1 (Ref.)	
≤ median	1.73 (0.49; 6.12)		1.61 (0.44; 5.86)	
> median	**5.08 (1.56; 16.52)**		**4.84 (1.47; 15.97)**	
Bone TLV		0.101		0.111
0	1 (Ref.)		1 (Ref.)	
>0	2.37 (0.90; 6.23)		2.31 (0.88; 6.10)	
Lymph node TLV		0.172		0.193
0	1 (Ref.)		1 (Ref.)	
≤ median	1.89 (0.57; 6.27)		1.77 (0.53; 5.95)	
> median	2.62 (0.95; 7.23)		2.57 (0.93; 7.10)	
Prostate TLV		0.123		0.121
0	1 (Ref.)		1 (Ref.)	
≤ median	1.20 (0.39; 3.73)		1.16 (0.37; 3.62)	
> median	**4.76 (1.34; 16.88)**		**4.79 (1.35; 17.00)**	

* Adjusted for age. Hazard ratios (HR) and 95% confidence intervals (95% CI) derived from Cox regression analyses. Analyses include patients from the biochemical recurrence cohort

## Discussion

This retrospective, single-centre study provides insight into the prognostic role of tumour volume quantified using [^18^F]PSMA-1007 PET/CT in patients with prostate cancer, both in primary staging and BCR. TLV was associated with OS, with distinct prognostic patterns in newly diagnosed patients and in those with BCR. In primary staging, the presence of bone metastases was the strongest predictor of poorer survival, whereas in BCR, high total TLV and high prostate TLV were associated with increased mortality risk.

Our study is related to recent work by Karpinski et al. (2024), updated in 2025, which demonstrated the prognostic utility of PSMA PET/CT findings using PROMISE criteria in predicting survival across different stages of prostate cancer (Karpinski et al. 2024, 2025). However, PROMISE does not yet integrate predictive modelling for OS, indicating an area for future research. Expanding the PROMISE framework to include prognostic parameters could further enhance its utility in clinical practice. The current study extends these findings by emphasizing the importance of specific tumour characteristics, such as TLV and the presence of metastases, which can be reliably assessed through PSMA PET/CT imaging.

Bone TLV showed a statistically significant impact on OS in the staging group but not in the BCR analysis. Given that bone metastases are associated with poor prognosis in prostate cancer (Liu et al. 2020), the limited number of patients with bone metastases in this study may have contributed to the lack of more significant findings. Another limitation of the analyses of bone metastasis is the risk of unspecific bone uptake with the [^18^F]PSMA-1007 tracer. Hence, there is a risk of misclassification of bone metastases, which may have caused a dilution of the true association (Seifert et al. 2023).

In the analyses of the primary staging group, patients with lymph node TLV above the median in the unadjusted model showed a significant association with OS (HR = 2.99). In the adjusted analysis a clear trend, although not significant, was observed, indicating that the presence and extent of lymph node involvement are critical for assessing prognosis. These findings underscore the importance of lymph node assessment in staging and treatment planning for prostate cancer, supporting previous studies that have highlighted the prognostic role of lymph node metastasis in prostate cancer outcomes (Daneshmand et al. 2004; Moris et al. 2016). Subdividing lymph node TLV by anatomical region would have produced very small groups with limited statistical power and unstable estimates and was therefore not performed in our study. Future studies with larger cohorts in which lymph node metastases are categorized by location could further investigate this, potentially revealing differences in OS between local and distant lymph node metastases.

In the primary staging and BCR subgroups, total, bone, lymph node, and prostate TLV showed clear differences on the impact on survival. In the BCR subgroup, TLV in bone and lymph nodes did not reach statistical significance, although the effect estimates suggested a possible association that may not have been detectable given the limited sample size. This subgroup had few observations which might have rendered the analyses underpowered to detect such an association. In addition, the discrepancy between the staging and BCR groups may reflect the different biological and clinical dynamics at play in patients with primary versus recurrent disease. For patients undergoing primary staging, TLV may be confined to the prostate and not be metastatic, meaning the disease can still be amenable to curative treatments like surgery or radiotherapy. Furthermore, in the staging group, patients with very high PSA-levels at diagnosis, typically over 100, are usually assessed with bone scans and CT at our centre, due to lower costs. This practice may have excluded patients with very high metastatic tumour burden in the primary staging group, which might have affected our data.

Additionally, 5–10% of prostate cancer do not express PSMA, meaning that no or very little uptake is seen on the PSMA PET/CT (Budaus et al. 2016). For this group, the PSMA PET/CT will not be a reliable prognostic factor (Rosenzweig et al. 2022) and other studies evaluating tumour volume in PSMA PET/CT have excluded these patients (Seifert et al. 2021). This may explain why this study found that absence of a prostate lesion was a worse prognostic factor compared to low TLV in the primary staging group.

Our results have important clinical implications, particularly in the context of personalized treatment for prostate cancer. PSMA PET/CT provides a more accurate assessment of disease burden compared to conventional imaging modalities like CT and bone scintigraphy, which are known to have lower sensitivity and specificity. Linking tumour volume, as detected by PSMA PET/CT, with survival outcomes could enhance the precision of treatment planning, allowing clinicians to better identify patients that might benefit from more aggressive or early interventions. For instance, patients with high TLV could be considered for treatment intensification, such as chemotherapy or androgen receptor pathway inhibitors (ARPIs), which have demonstrated efficacy in prolonging survival in metastatic prostate cancer (Emmett et al. 2025). Additionally, integrating TLV as a prognostic marker in clinical trials could help refine patient selection, improving the accuracy of trial outcomes (Emmett et al. 2025; Attard et al. 2023; Kyriakopoulos et al. 2018).

Strengths in our study include a robust sample size (*n* = 282) and a long median follow-up period of 5 years. However, it is important to acknowledge some limitations. The study is retrospective, which limits the ability to control for biases related to patient selection and treatment heterogeneity. Furthermore, the relatively small number of patients with bone and lymph node metastasis impaired the ability to fully assess the prognostic role, a limitation that could be addressed in future studies with larger cohorts. Another limitation is the lack of data regarding the impact of specific treatments, which may confound the relationship between tumour volume and OS. Furthermore, a high TLV in the lymph nodes could also indicate bone metastases and this could be the principal leading factor of death in this group, since bone metastasis are considered a worse prognostic factor than lymph nodes (Gandaglia et al. 2015). Finally, additional prognostic variables (e.g. treatment, Gleason score, PSA) were not included in multivariate analyses due to the small number of events.

Studies exploring the relationship between PSMA PET/CT findings and treatment-specific outcomes, such as progression-free survival or response to specific therapies, would be valuable in further clarifying the role of PSMA PET/CT as an imaging biomarker guiding treatment decisions.

## Conclusions

This study demonstrates that TLV, as measured by [^18^F]PSMA-1007 PET/CT, is associated with OS in prostate cancer patients, with different prognostic patterns in newly diagnosed patients and in those with BCR. The findings suggest that PSMA PET/CT could serve as a valuable tool in predicting long-term outcomes and informing treatment strategies. Although further research is needed to validate these results and expand the understanding of PSMA PET/CT’s prognostic potential, this study represents a step forward in the integration of advanced imaging technologies into prostate cancer care. By improving disease stratification and offering more personalized treatment options, PSMA PET/CT has the potential to significantly enhance prediction of patient outcomes in prostate cancer management.

## Electronic supplementary material

Below is the link to the electronic supplementary material.

Supplementary Material 1: Figure 1. Survival curves for the whole cohort by total, lymph node, bone and prostate tumour volume. Tumour volume divided in none, below median, and above median for total TLV, lymph node TLV and prostate TLV. Bone TLV presented as not detectable or detectable.

Supplementary Material 2

## Data Availability

All data are archived according to the Swedish Act concerning the Ethical review of Research Involving Humans to attain confidentiality and are available on reasonable request.

## Abbreviations

PSMA: PET/CT Prostate-specific membrane antigen positron emission tomography/computed tomography
TLV: Total lesion volume
OS: Overall survival
BCR: Biochemical recurrence
BS: Bone scintigraphy
CI: Conventional imaging
SUVmean: Standardized PSMA PET/CT Analysis and Reporting Consensus (SPARC) mean and maximum Standardized uptake value mean
SUVmax: Standardized uptake value max
PROMISE: Prostate Cancer Molecular Imaging Standardized Evaluation
HR: Hazard ratios
CI: Confidence intervals
ARPIs: Androgen receptor pathway inhibitors
