# Supplementary figures and images for: Prognostic value of tumour volume based on [18F]PSMA-1007 PET/CT in prostate cancer
Source: EJNMMI Rep. 2026 Mar 13;10(1):9. doi: 10.1186/s41824-026-00292-w (PMC12982702; doi:10.1186/s41824-026-00292-w)

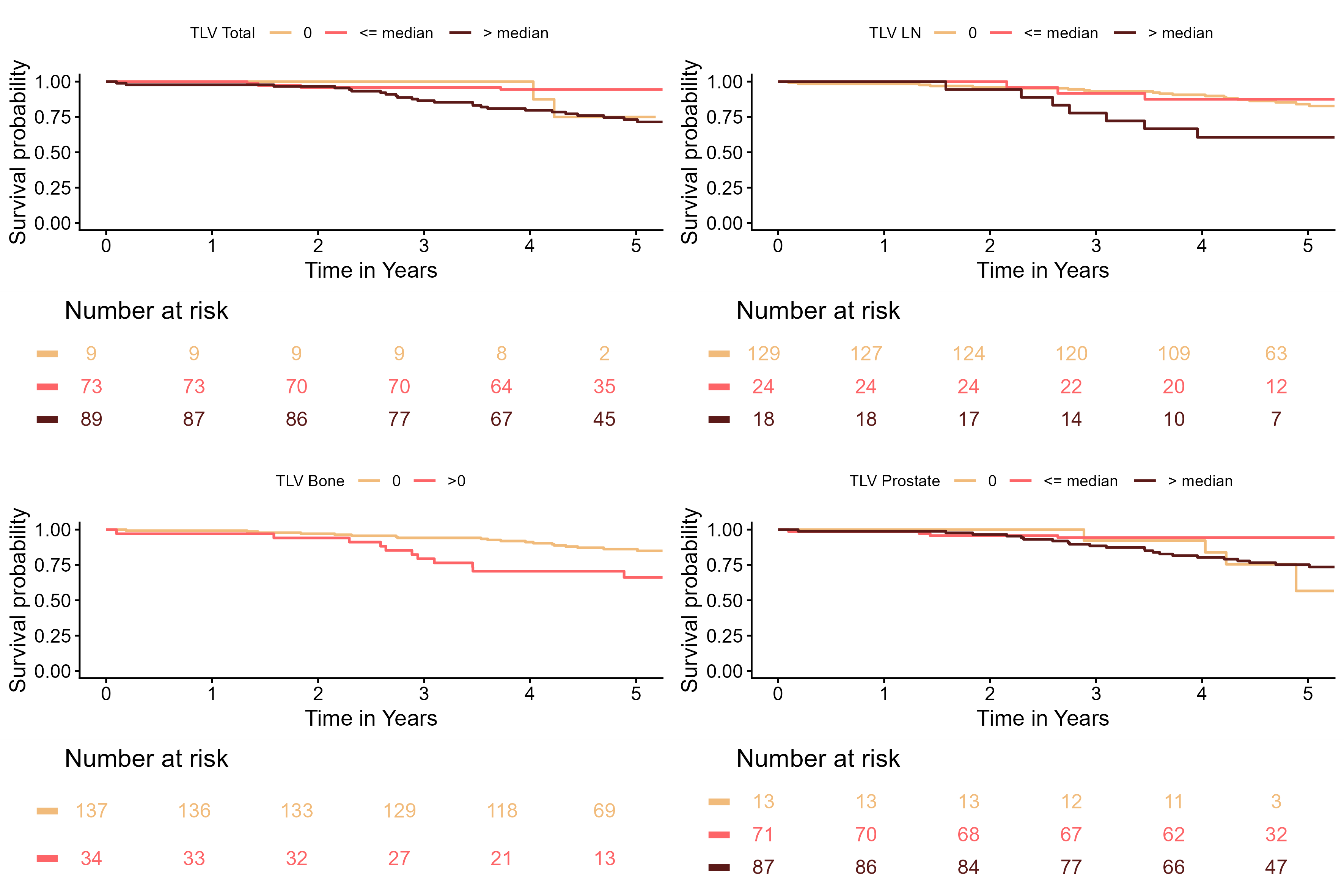

Supplement: Supplementary file 1 — Supplementary Material 1: Figure 1. Survival curves for the whole cohort by total, lymph node, bone and prostate tumour volume. Tumour volume divided in none, below median, and above median for total TLV, lymph node TLV and prostate TLV. Bone TLV presented as not detectable or detectable. [file 41824_2026_292_MOESM1_ESM.tiff]
