# Supplementary material for: Prognostic value of tumour volume based on [18F]PSMA-1007 PET/CT in prostate cancer
Source: EJNMMI Rep. 2026 Mar 13;10(1):9. doi: 10.1186/s41824-026-00292-w (PMC12982702; doi:10.1186/s41824-026-00292-w)
Supplement: Supplementary file 2 — Supplementary Material 2 [file 41824_2026_292_MOESM2_ESM.docx]

Supplementary table 1: Hazard ratios and 95% confidence intervals for overall survival including all patients

|  | **Unadjusted** | | **Adjusted^*^** | |
| --- | --- | --- | --- | --- |
| **Variable** | **HR (95% CI)** | **p-value** | **HR (95% CI)** | **p-value** |
| Total TLV |  | <0.001 |  | <0.001 |
| 0 | 1 (Ref.) |  | 1 (Ref.) |  |
| ≤ median | 0.80 (0.29; 2.21) |  | 0.70 (0.25; 1.94) |  |
| > median | **2.89 (1.21; 6.90)** |  | **2.43 (1.01; 5.88)** |  |
| Bone TLV |  | 0.003 |  | 0.008 |
| 0 | 1 (Ref.) |  | 1 (Ref.) |  |
| >0 | **2.56 (1.42; 4.61)** |  | **2.34 (1.30; 4.24)** |  |
| Lymph node TLV |  | 0.023 |  | 0.044 |
| 0 | 1 (Ref.) |  | 1 (Ref.) |  |
| ≤ median | 1.19 (0.52; 2.71) |  | 1.03 (0.45; 2.38) |  |
| > median | **2.62 (1.38; 4.98)** |  | **2.35 (1.23; 4.49)** |  |
| Prostate TLV |  | 0.006 |  | 0.010 |
| 0 | 1 (Ref.) |  | 1 (Ref.) |  |
| ≤ median | 0.49 (0.21; 1.15) |  | 0.47 (0.20; 1.09) |  |
| > median | 1.64 (0.87; 3.07) |  | 1.46 (0.78; 2.76) |  |

^*^Adjusted for age. Hazard ratios (HR) and 95% confidence intervals (95% CI) derived from Cox regression analyses. Analyses combine patients from the primary staging cohort and the biochemical recurrence cohort.
